
JOURNAL INFORMATION
==============================
NLM Title Abbreviation: Wirtschaftsdienst
Journal ID: Wirtschaftsdienst
NLM Journal ID: 101086612

Wirtschaftsdienst (Hamburg, Germany : 1949)

ISSN: 0043-6275

ARTICLE INFORMATION
==============================
PMCID: PMC7441476
PMID: 32843773
DOI: 10.1007/s10273-020-2707-2
Article ID: 2707
Article version: 1
Subjects: Kurz Kommentiert

Union auf Pump

EU Special Summit: Union on credit

Büttner Thiess Thiess.Buettner@fau.de

LS für Volkswirtschaftslehre, Friedrich-Alexander Uni. Nürnberg-Erlangen, Lange Gasse 20, 90403 Nürnberg, Deutschland
Electronic publication date: 2020 Aug 21
Print publication date: 2020
Volume: 100
Issue: 8
First page: 565
Last page: 565
Copyright: © Der/die Autor(en) 2020
License: Open Access Dieser Artikel wird unter der Creative Commons Namensnennung 4.0 International Lizenz veröffentlicht, welche die Nutzung, Vervielfältigung, Bearbeitung, Verbreitung und Wiedergabe in jeglichem Medium und Format erlaubt, sofern Sie den/die ursprünglichen Autor(en) und die Quelle ordnungsgemäß nennen, einen Link zur Creative Commons Lizenz beifügen und angeben, ob Änderungen vorgenommen wurden.
Die in diesem Artikel enthaltenen Bilder und sonstiges Drittmaterial unterliegen ebenfalls der genannten Creative Commons Lizenz, sofern sich aus der Abbildungslegende nichts anderes ergibt. Sofern das betreffende Material nicht unter der genannten Creative Commons Lizenz steht und die betreffende Handlung nicht nach gesetzlichen Vorschriften erlaubt ist, ist für die oben aufgeführten Weiterverwendungen des Materials die Einwilligung des jeweiligen Rechteinhabers einzuholen.
Weitere Details zur Lizenz entnehmen Sie bitte der Lizenzinformation auf http://creativecommons.org/licenses/by/4.0/deed.de.
License URL: https://creativecommons.org/licenses/by/4.0/

issue-copyright-statement: © The Author(s) 2020

==============================
DOI: 10.1007/s10273-020-2707-2

EU-Sondergipfel

### Union auf Pump

Die Beschlüsse des Sondergipfels des Europäischen Rates vom Juli 2020 sind verschiedentlich als „historisch“ bezeichnet worden. In der Tat sind Umfang der bereitgestellten Mittel und Art der Finanzierung außerordentlich. Allerdings werden Superlative in Europa gern bemüht. So sollten die Beschlüsse des Lissabon-Gipfels vor 20 Jahren dazu führen, Europa zum „wettbewerbsfähigsten und dynamischsten Wirtschaftsraum in der Welt“ werden zu lassen. Im Dezember 2019 sollte der „Green Deal“, Europas „Mann-auf-dem-Mond-Moment“ herbeiführen. Wie sind die Beschlüsse also zu bewerten?

Zwei Sachverhalte sind zu unterscheiden: erstens die Beschlüsse zum EU-Haushalt für die Budgetperiode 2021 bis 2027, zweitens die Beschlüsse über Krisenhilfen. Das EU-Budget sah in der auslaufenden Budgetperiode ein Volumen von 960 Mrd. Euro vor. Dies entsprach bei den Verhandlungen 2013 in der Erwartung etwa 1 % des Bruttonationaleinkommens (BNE) der EU der 28. Der neue Haushalt soll nun mit 1.074 Mrd. Euro ausgestattet werden – nach Brüsseler Berechnungsweise etwa 1,05 % des BNE der EU der 27. So konnte die EU zumindest teilweise den Budgeteinbruch wegen ihres Bedeutungsverlustes im Zuge des „Brexit“ abwehren. Inhaltlich machen Agrarpolitik und Strukturhilfen wie schon in der Vergangenheit rund zwei Drittel des Budgets aus. Es bleibt also dabei, dass der Brüsseler Haushalt das Subsidiaritätsprinzip verletzt und eben nur am Rande Güter und Dienste finanziert, die einen echten europäischen Mehrwert entfalten.

Außergewöhnlich an den Beschlüssen sind die Krisenhilfen im Rahmen des Sonderpakets „Next Generation EU“ (NGEU), das ein Volumen von 750 Mrd. Euro umfasst. Der größte Einzelposten in diesem Paket (672 Mrd. Euro) sind Transfers und Darlehen für die Bekämpfung der Folgen der COVID-19-Pandemie. Die Verteilung der Mittel soll zu 70% nach wirtschaftlichen Leistungsindikatoren erfolgen – dem BIP pro Kopf des Jahres 2019 und der Arbeitslosigkeit in den Jahren 2015 bis 2019. Nur für die verbleibenden 30 % ist teilweise eine Berücksichtigung der Stärke des aktuellen wirtschaftlichen Einbruchs vorgesehen. Neu am NGEU-Paket ist neben der Entscheidung für Transfers auch die Finanzierung. Sie soll nicht wie beim Haushalt aus den laufenden EU-Eigenmitteln erfolgen, sondern durch die Ausgabe von Anleihen. Zwar hat die EU z. B. zur Finanzierung der Europäischen Investitionsbank bereits Anleihen ausgegeben. Jetzt ist allerdings vorgesehen, auch die nicht rückzahlbaren Transfers im Umfang von rund 384 Mrd. Euro durch Verschuldung zu finanzieren.

Mit Ausnahme des EU-Parlaments, das in den Hintergrund gedrängt wird, schafft das NGEU-Paket scheinbar nur Gewinner. Die aktuelle EU-Kommission kann über einen enormen Mittelzuwachs verfügen, die Mitgliedsländer werden umfangreiche Finanzhilfen erhalten, die überwiegend nicht zurückgezahlt werden müssen, und die Beiträge zum EU-Haushalt bleiben innerhalb der kommenden sieben Jahre unberührt. Möglicherweise bleiben auch die Steuerzahler zunächst verschont. Die Vorstellung, dass Finanzhilfen in dieser Größenordnung nicht auch massive Belastungen mit sich bringen, ist aber eine Illusion.

Natürlich werden die Mitgliedsländer höhere Beiträge leisten müssen und die Steuerzahler in die Pflicht nehmen. Länder mit schwachen Wirtschaftszahlen erhalten nur vorübergehend höhere Transfers – die strukturellen Finanzprobleme sind damit nicht gelöst. Die Verteilungskonflikte werden durch den Einstieg in die Transferunion an Bedeutung gewinnen und der Fokus der Mitgliedsländer auf die Nettosalden wird befeuert. Zwar wird von politischer Seite gern betont, dass die Bereitstellung von Krisenhilfen in der Pandemie als ein Zeichen der Solidarität die EU stärkt. Allerdings verteilt das NGEU-Paket Finanzmittel überwiegend nicht nach der Betroffenheit von der Pandemie, sondern nach wirtschaftlichen Eckdaten aus der Zeit vor der aktuellen Krise.

Dass das NGEU-Paket durch Anleihen und nicht durch Eigenmittel finanziert wird, zeigt in erster Linie, dass die EU ein ungelöstes Finanzierungsproblem hat. Hierin einen „Hamiltonian Moment“, eine fundamentale Änderung der Rahmenbedingungen der EU hin zur Errichtung einer zentralen staatlichen Ebene in Europa zu sehen, wäre grundfalsch. Die von Alexander Hamilton 1790 betriebene Nationalisierung der Staatsschulden der US-Bundesstaaten ging mit einer Übertragung der wichtigsten Steuerquelle auf die Zentralregierung einher. Die EU wird aber weiterhin durch Beiträge und nicht aus Steuerquellen finanziert.

Möglicherweise wird der Zusammenhalt der EU der 27 neben dem 750 Mrd. Euro teuren Solidaritätssignal aber auch dadurch gestärkt, dass die Austrittskosten eines Mitgliedslandes aus der EU steigen: in der „Exit Bill“, die einem künftig aussteigenden Mitgliedsland präsentiert würde, dürfte die anteilige Schuldenlast mit aufgenommen werden.

Thiess Büttner
Friedrich-Alexander-Universität Erlangen-Nürnberg
thiess.buettner@fau.de

© Der/die Autor(en) 2020. Open Access: Dieser Artikel wird unter der Creative Commons Namensnennung 4.0 International Lizenz (https://creativecommons.org/licenses/by/4.0/deed.de) veröffentlicht.

Open Access wird durch die ZBW – Leibniz-Informationszentrum Wirtschaft gefördert.
